# A study protocol to investigate the relationship between dietary fibre intake and fermentation, colon cell turnover, global protein acetylation and early carcinogenesis: the FACT study

**DOI:** 10.1186/1471-2407-9-332

**Published:** 2009-09-18

**Authors:** Bernard M Corfe, Elizabeth A Williams, Jonathan P Bury, Stuart A Riley, Lisa J Croucher, Daphne YL Lai, Caroline A Evans

**Affiliations:** 1 Department of Oncology, University of Sheffield, The Medical School, Beech Hill Road, Sheffield, S10 2RX, UK; 2 Department of Gastroenterology, Northern General Hospital, Herries Road, Sheffield, UK; 3 Department of Chemical and Process Engineering, University of Sheffield, Mappin St, Sheffield, S1 3JD, UK; 4 Arthritis Research Campaign, St Mary's Gate, Chesterfield, S41 7TD, UK; 5 Department of Geography, University of Sheffield, Sheffield, S10 2TN, UK

## Abstract

**Background:**

A number of studies, notably EPIC, have shown a descrease in colorectal cancer risk associated with increased fibre consumption. Whilst the underlying mechanisms are likely to be multifactorial, production of the short-chain fatty-acid butyrate fro butyratye is frequently cited as a major potential contributor to the effect. Butyrate inhibits histone deacetylases, which work on a wide range of proteins over and above histones. We therefore hypothesized that alterations in the acetylated proteome may be associated with a cancer risk phenotype in the colorectal mucosa, and that such alterations are candidate biomarkers for effectiveness of fibre interventions in cancer prevention.

**Methods an design:**

There are two principal arms to this study: (i) a cross-sectional study (FACT OBS) of 90 subjects recruited from gastroenterology clinics and; (ii) an intervention trial in 40 subjects with an 8 week high fibre intervention. In both studies the principal goal is to investigate a link between fibre intake, SCFA production and global protein acetylation. The primary measure is level of faecal butyrate, which it is hoped will be elevated by moving subjects to a high fibre diet. Fibre intakes will be estimated in the cross-sectional group using the EPIC Food Frequency Questionnaire. Subsidiary measures of the effect of butyrate on colon mucosal function and pre-cancerous phenotype will include measures of apoptosis, apoptotic regulators cell cycle and cell division.

**Discussion:**

This study will provide a new level of mechanistic data on alterations in the functional proteome in response to the colon microenvironment which may underwrite the observed cancer preventive effect of fibre. The study may yield novel candidate biomarkers of fibre fermentation and colon mucosal function.

**Trial Registration:**

Trial Registration Number: **ISRCTN90852168**

## Background

Since Burkitt's original observations on the inverse correlation between fibre (non-starch polysaccharides and resistant starch) intake and prevalence of colorectal cancer [1], a wide range of studies have addressed this relationship and the possible mechanisms by which fibre may protect against bowel cancer. Recent meta-analyses find a strong evidence base to support consumption of fibre-containing foods for prevention of several cancers [2], and the majority of studies in this area are supportive. There are exceptions, however, and two RCT studies, published in 2000, failed to demonstrate a protective effect [3,4]. These controversial findings have been the subject of several commentaries [5,6]. Potential explanations for this conflicting data include: differences between US and EU assays for fibre, different baseline levels of intake and the limitations of adenoma recurrence as a model for primary colorectal cancer prevention.

There are several mechanisms proposed for fibre's proposed cancer-preventive properties. These include dilution of luminal contents; reduction in transit time, which together will reduce exposure of the mucosa to luminal toxin; adsorbtion of bile acids; and production of protective short chain fatty acids (SCFAs: principally acetate, propionate and butyrate) through fermentation of fibre by endosymbiotic bacteria. Studies in rats treated with a colorectal carcinogen, have demonstrated a variable protective effect of different dietary fibre substrates and have linked this with changes in the luminal SCFA profile [7]. Gibson et al for example found that when rats consumed a diet with cellulose, a non-fermentable fibre, as principle fibre source, little protection from DMH-induced carcinogenesis was afforded. Oat-derived fibre, an acutely fermentable fibre which is rapidly turned over to SCFA in the caecum, but yields lower levels of SCFA in the distal colon and rectum, provided improved protection, but maximal protection was conferred by the more weakly fermentable wheat fibre, which yielded higher levels of SCFA in the distal colon and rectum. The study analysed SCFA levels in rats' stools on each regimen and found that the strongest correlation with cancer prevention in this model occurred on diets which gave maximal elevation of faecal butyrate. Not surprisingly this data has led to a resurgence of interest in the actions of butyrate.

Roediger [8] was first to show that butyrate is the preferred metabolite of colon epithelial cells. In his studies, primary epithelial cells from rat colon were incubated with labelled glucose and labelled butyrate. Butyrate was found to be metabolised in preference to glucose, which is available to colonocyte in vivo through the vasculature. The use of butyrate as an energy souce is inefficient (by comparison with glucose) and it has been suggested that this represents an evolutionary adaptation to recover the maximum energy available from the high-fibre diets consumed by our paleolithic ancestors.

The effect of butyrate on cells grown in vitro is to drive both cell cycle arrest and apoptosis. Both of these alterations in cell fate occur at concentrations of butyrate readily achieved in the colon lumen through fibre fermentation. Cell cycle arrest has variously been reported as G1 arrest, G2 arrest and mitotic bypass [9-11]. Several reports have shown that the apoptosis triggered by butyrate in vitro is associated with dysregulation of Bcl2 family proteins especially upregulation of BAK and downregulation of BclxL [12-14], rather than cellular damage.

These in vitro data contrast with studies on the in vivo or ex vivo effects of butyrate. Takayama's studies investigating the effect of increasing fibre intake after restriction, using a variety of animal models, have shown that switching to a high fibre diet is associated with an increase in colon crypt length, cellularity and proliferation [15-17]. Hass [18] used ex vivo guinea pig colon mucosa in an Ussing chamber model and monitored rates of cell death. When tissue was maintained in an osmotically balanced chamber, widespread cell death was found on the epithelium and this was associated with up-regulation of Bax. When butyrate was added to the chamber, however, there was reduced cell death and no Bax upregulation. Furthermore, studies of diversion colitis show that widespread cell death occurs after diversion of the faecal stream and loss of luminal content [19]. This condition may be ameliorated by butyrate enema [20].

Recent studies have shown that elevation of luminal SCFA causes no direct increase in levels of epithelial apoptosis [21,22], but causes a significant increase in the level of apoptosis after a genotoxic challenge. These in vivo data are suggestive of a model whereby butyrate's antineoplastic action is not in the induction of apoptosis per se, but through sensitization of cells to damage. The observations made in vitro that butyrate elevated levels of pro-apoptotic Bcl2 family proteins, and downregulated their anti-apoptotic counterparts could be predicted to sensitize cells in precisely this way and we have recently proposed this as a model [15].

How might butyrate alter cell functionality in this way? Although recognised as a metabolite, butyrate is also a potent inhibitor of histone deacetylases (HDACs) - [23]. HDACs are primarily recognised as one part of the regulatory mechanism for governing histone acetylation levels in concert with their agonist enzymes the histone acetyl transferases (HATs). The acetylation state of histones is thought to be a potent governor of gene transcription at both a specific and regional level of the chromatin. A number of publications have shown widespread alteration in gene expression after treatment of cells in vitro with butyrate, indicating as much as 10% of genes may be affected by butyrate either directly or indirectly. However more recently several groups have identified other acetyl proteins in the nucleus and cytosol, and HDAC activities have been found in both cellular compartments [24,25]. Amongst the acetyl proteins identified are nuclear structural proteins, transcription factors including p53, Sp1, Sp3 [26] and structural proteins including tubulin and cytokeratins [27,28]. Our own preliminary findings using pan-specific antiacetyl lysine antibodies indicate tens or hundreds of acetyl proteins in cell lines (Leech & Corfe, unpublished). Acetylation has been proposed as being as important as phosphorylation in the regulation of protein function [29]. This is reinforced by the observation that HATs and HDACs are frequently mutated in cancer, which may lead to an alteration in the acetylation landscape of the cell permissive for cancer progression.

Taken together these data allow us to generate an hypothesis that i) colorectal carcinogenesis will be associated with an alteration in global protein acetylation, ii) reduced levels of butyrate will cause alterations in global protein acetylation, which may also be permissive for colorectal cancer progression, iii) that elevation of fibre levels and consequent butyrate levels may reduce or reverse these processes and restore a "normal" profile of protein acetylation.

## Methodsand design

### Overall Aim of the study

To determine if there is a link between global protein acetylation, fibre intake and fermentation, and colorectal carcinogenesis.

#### Primary Aims

1. To undertake a cross-sectional study of global protein acetylation profiles in normal subjects and those with colonic polyps and colorectal cancer. This study arm is named FACT OBS.

2. To determine, in the same clinical groups, the relationship between global protein acetylation profile and faecal SCFA levels.

3. To undertake a fibre supplementation study in morphologically normal GI patients and patients with colonic polyps to determine the effect of fibre supplementation on the global acetylation profile. This study arm is named FACT INT.

#### Secondary Aims

1. To investigate in the above groups whether there is an alteration in crypt proliferation index in response to carcinogenesis and to fibre.

2. To investigate in the above groups whether there is an alteration in the apoptotic index in response to carcinogenesis and to fibre.

3. To investigate in the above groups whether this is an alteration in expression of key apoptotic regulators in response to carcinogenesis and to fibre.

4. To establish the effect of bowel cleansing preparations on the parameters measured under the secondary aims. This study arm is named FACT VAL.

5. Evaluation of the EPIC Food Frequency Questionnaire as a proxy measure of faecal SCFA.

#### Ethics

Ethics committee approval was obtained from the North Sheffield Research Ethics Committee prior to recruiting (Reference number: 06/Q2308/93)

#### Patient Recruitment and sample collection

##### FACT OBS recruitment

Recruitment targets were 30 normal, 30 polyp and 30 cancer patients. Patients were primarily recruited via outpatient clinics and through patient databases at Sheffield's Northern General Hospital and Royal Hallamshire Hospital. All patients were attending for diagnostic colonoscopy and patient and researchers were unaware of the diagnosis at time of recruitment and consent. Up to 10 biopsies were collected at endoscopy from these patients. Biopsies were collected with a Radial Jaw 4 2.8 mm forceps (see table 1). Two weeks post-endoscopy, patients were asked to provide a stool sample and completed a Food Frequency Questionnaire (FFQ).

**Table 1:** Position and uses of biopsies taken during endoscopy for the FACT OBS and FACT INT arms of this study.

Site	Biopsies	Purpose
**FACT OBS**		

Mid-sigmoid	1	Whole mount
	2	Immunohistochemistry
	3	Proteomics
	4	Proteomics

Contralateral	1	Immunohistochemistry
	2	Proteomics
	3	Proteomics

Lesion	1	Immunohistochemistry
	2	Proteomics
	3	Proteomics

**FACT INT**		

Mid-sigmoid	1	Whole mount
	2	Immunohistochemistry
	3	Immunohistochemistry
	4	Proteomics
	5	Proteomics
	6	Proteomics
	7	Proteomics
	8	Proteomics
	9	Proteomics
	10	Proteomics

##### FACT VAL recruitment

Recruitment of patients to the FACT VAL arm of the study was from the same routes as for the FACT OBS arm. Patients indicating a willingness to return for a repeat flexible sigmoidoscopy without bowel preparation were recruited specifically to this arm of the study. The recruitment target for this arm was 6 patients with a first procedure following preparation with Kleanprep and 6 patients with a first procedure following preparation with Picolax.

##### FACT INT recruitment

Recruitment target for the FACT INT study is 20 normal and 20 polyp subjects consuming an habitually low fibre diet. Patients will be recruited via the outpatients clinics for the bowel screening programme, Northern General Hospital, Sheffield. The biopsy protocol is summarised in Table 1. Stool samples, FFQ and a four day food diary will be collected circa 2 weeks after the endoscopy. Subjects will be asked to comply with a high fibre diet for an eight week period (including a 2 week ramp phase). Stool sampling and a further food diary will be collected at the end of the 8 week intervention, patients will then return for a second endoscopy with the same biopsying protocol.

##### Power calculation and sample size

There are no previous studies investigating addressing alteration in global protein acetylation *in vivo *and therefore no data upon which to base a power calculation.

#### Inclusion and Exclusion Criteria

##### Inclusion criteria

Male subjects (FACT OBS only), Aged > 40, Low habitual NSP and RS consumption (FACT INT only), Low level of faecal butyrate BMI 20-29.

##### Exclusion criteria

Female (FACT OBS only), Cancer (FACT INT only), Habitual consumers of a diet high in NSP and RS, Smokers, Type 2 diabetics, Dieters, Inflammatory Bowel Disease.

#### High Fibre intervention

The primary goal of the intervention arm is to elevate colonic SCFA levels, which will be monitored through analysis of faecal SCFA. Elevation of fermentable fibre intake is a rapid and amenable mechanism to achieve this goal. Patients recruited to the FACT INT arm will be offered a range of high-fibre foods, including switch to wholemeal bread, fruit, dried fruit snacks, from a basket of options identified by the research team (Additional File 1). Foods will be ordered by the researcher and delivered by a supermarket home delivery to the patients' homes. Compliance will be estimated and supported through interim telephone calls and 24 hr recall estimates.

#### Primary outcome measure

##### Development of a methodology for studying global protein acetylation in biopsy samples and proof of principle studies

It is the goal of this study to link alterations in global proteins acetylation in the colon mucosa to levels of butyrate, the most potent HDACi produced though colonic fermentation of fibre. In order to determine levels of butyrate and other SCFA, the stool sample collected from patients will be weighed and extracted freshly (within 3 hr of production) using a standard procedure [29] to yield SCFA. The absolute level of levels of each SCFA will be determined by gas chromatography ion collaboration with Prof Chris Seal, University of Newcastle upon Tyne. The acetylated proteome will be analysed by a method reported elsewhere (Croucher et al., in preparation), modified from a published protocol for analysis of global protein acetylatyion [30]. In brief, tissue samples will be lysed, soluble protein extracted and acetyl proteins immunoprecipitated before separation by 2d gel electrophoresis. Owing to the numbers of biopsies' worth of immunoprecipitate required for a 2d gel (6-8 biopsies), a pooling strategy will be required for the FACT OBS analysis. Patients will be pooled into deciles within pathology groups (i.e. deciles within adenoma group, cancer group, normal group) according to faecal butyrate levels, and all biopsies in each pool will be used for a single 2d gel.

###### Pooling strategy for FACT INT

As larger numbers of biopsies are being taken from a single region of the colon per patient for the FACT INT study, no further pooling will be required.

###### Analysis of gels and difference discovery

The software of choice for analysis, quantitation of 2 d gels and for identification of differences between gels will be Samespots [31].

###### Residual proteome

The residual proteome (i.e. proteins not bound on the acetyl-IP column) will be separated and analysed using an iTRAQ workflow [32] to investigate comprehensively possible differences between the epithelial proteome in response to carcinogenesis and to SCFA.

#### Secondary outcome measures

##### Alteration in crypt cell proliferation in i) carcinogenesis; ii) response to SCFA; iii) in response to elevation of SCFA

Several methodologies/measures are available for assessing crypt cell proliferation rates. These include immunohistochemical approaches on formalin-fixed, paraffin-embedded (FFPE) sections and whole mount analysis. FFPE sections may be probed with a number of antibodies to give a measure of cell proliferation. Antibodies to the Ki67 antigen cross-react with all cells actively in cycle, irrespective of the stage in cell cycle. Some commentators suggest that immunohistochemical approaches are insufficient to measure accurately mitosis as they depend on evaluation within a cross-section whereas mitoses will occur in the 3d structure of the crypt [33]. Whole mount analysis, involving the fixation of biopsies, followed by staining with Feulgen's reagent and scoring mitosis in the full depth of the crypt offers an alternative approach [34].

In this study we will use both Ki67 and whole mount/Felugen's as assays of crypt cell proliferation. Our preliminary findings show correlation between both indices and suggest that Ki67 may be an adequate general index of proliferation [35].

We will assess the relationship between fibre intake, SCFA levels, butyrate levels, associated pathology and each of the proliferation indices in samples collected in the FACT OBS study. We will assess whether differences observed attributed to either fibre/SCFA or pathology can be reverted by elevation of fibre intake and faecal SCFA level with samples collected in the FACT INT study.

##### Alteration in mucosal apoptosis in i) carcinogenesis; ii) response to SCFA; iii) in response to elevation of SCFA

Animal models have suggested that elevated fibre intake and fermentation alone do not alter levels of background apoptosis in the colon mucosa in rats. There are few if any studies addressing directly whether apoptotic rates are altered in response to fibre intakes/SCFA levels in humans. Several assay methods are available for the scoring of apoptosis, including antibodies to protein cleavage products specifically generated during apotosis. The M30 antibody recognises a neoepitope produced through cleavage of cytokeratin 18, and the CC3 antibody recognises the cleaved, activated form of apoptosis-specific protease caspase 3.

As described above for the proliferation indices, we will assess the relationship between fibre intake, SCFA levels, butyrate levels, associated pathology and apoptositic index in the FACT OBS study and establish wther regression to normality can be achieved through elevation of fibre intakes through the FACT INT study.

##### Alteration in mucosal apoptotic regulators in i) carcinogenesis; ii) response to SCFA; iii) in response to elevation of SCFA

Despite elevation of SCFA not driving increased apoptosis in the colon mucosa, there is a suggestion that the mucosa is sensitized to damage and will show an improved apoptotic response to cytotoxic insult. Taken together with data from in vitro studies showing apoptosis driven by butyrate is associated with dysregulation of Bcl2 family proteins, we hypothesize that although no particular finding may be made with the direct apoptosis assays, elevated SCFA levels may directly and measurably alter Bcl2 family expression.

Of particular interest are the pro-apoptotic members of the family, Bax and BAK. We will determine levels of expression using semi-quantitative immunohistochemical approaches. Bax, BAK and the anti-apoptotic protein Bcl2 will be determined in the FACT OBS and FACT INT studies.

## Discussion

The study aims to investigate for the first time the effects of fibre intake and disease pathology on global protein acetylation, and to link this data to commonly used measures of cell fate (cell division and cell death) whose derangement is a hallmark of cancer [36]. The approaches developed may yield novel biomarkers of either or both of fibre consumption or SCFA production and of the earliest stages of carcinogenesis. By establishing the potential for such biomarkers to revert to normality and linking these reversions to cellular events on the proliferative and apoptotic pathways we may in the medium term develop improved approaches to the promotion of colon health.

## Competing interests

The authors declare that they have no competing interests.

## Authors' contributions

BMC Conceived the project, directs the overall project, directed the proteomics method development and wrote the manuscript; EAW contributed to the study design, directs recruiting and nutritional analysis; JPB contributed to study design, directs immunohistochemical methods and analysis; SAR is the clinical lead for the study, contributed to the study design, undertakes endoscopy and directs clinical procedures; LJC developed methods for separation of acetyl proteins and undertakes immunohistochemical analysis; DYLL undertakes patient recruiting, intervention strategy and nutritional analysis; CAE conceived and undertakes quantitative proteomic analysis. All authors read and approved the final manuscript.

## Pre-publication history

The pre-publication history for this paper can be accessed here:

http://www.biomedcentral.com/1471-2407/9/332/prepub

## Supplementary Material

Additional file 1 Food choice form developed for high fibre foods offered as supplements in the FACT INT arms of this study. Click here for file
