# Supplementary material for: A study protocol to investigate the relationship between dietary fibre intake and fermentation, colon cell turnover, global protein acetylation and early carcinogenesis: the FACT study
Source: BMC Cancer. 2009 Sep 18;9:332. doi: 10.1186/1471-2407-9-332 (PMC2751778; doi:10.1186/1471-2407-9-332)
Supplement: Additional file 1 — Food choice form developed for high fibre foods offered as supplements in the FACT INT arms of this study. [file 1471-2407-9-332-S1.DOC]

**Food choice for FACT Study**

**FACT:**

**Fibre for Colon Health**


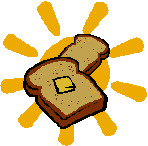


**Please mark √ for foods you wish to include in the high-fibre food basket**.

| **Cereals**  **(We recommend you to select > 1 type of cereal)** |  |  | **Salads** |  |
| --- | --- | --- | --- | --- |
| All-bran ………………………. |  |  | Bagged salad …………………….. |  |
| Shredded wheat ………………. |  |  | Tomatoes …………………………. |  |
| Bran flakes ………………………. |  |  | Cherry Tomatoes ………………… |  |
| Weetabix ………………………… |  |  |  |  |
| Muesli ……………………………. |  |  | **Prepared fruits** |  |
| Fruit & Fibre ……………………….. |  |  | Mango ………………………….. |  |
| Oat porridge ……………………….. |  |  | Fruit salad ……………………… |  |
|  |  |  |  |  |
| **Bread/Rice/Pasta**  **(* Essential for food basket)** |  |  | **Single fruits** |  |
| Crispbread, rye. .……………... |  |  | Avocado ……………………. |  |
| Pasta ……………………………… |  |  | Pear …………………………… |  |
| ***Wholemeal bread** …………….. | **√** |  | Orange …………………………. |  |
| Granary bread …………………….. |  |  | Apple ……………………………. |  |
| Brown rice ……………………………. |  |  | Raspberries ……………………. |  |
|  |  |  | Banana ……………………………. |  |
| **Prepared Vegetables** |  |  | Grapes ……………………………. |  |
| Baked beans in tomato sauce …. |  |  | Tomato juice ……………………… |  |
| Red kidney beans …………... |  |  |  |  |
| Peas, canned ……………………. |  |  | **Dried fruits/Nuts** |  |
| Broccoli Florets ………………………. |  |  | Figs, ready-to-eat ……….. |  |
| Cabbage Medley …………………….. |  |  | Apricots, ready–to-eat ….. |  |
| Carrot Batons ……………………… |  |  | Prunes, ready-to-eat ………. |  |
| Carrot/Cauliflower/Broccoli ………. |  |  | Almonds ………………………… |  |
| Sliced Runner Beans ……………….. |  |  | Hazelnuts ………………………… |  |
|  |  |  | Peanuts …………………………… |  |
| **Single Vegetables** |  |  | Mixed nuts ………………………… |  |
| Carrots ……………………………… |  |  | Brazil nuts ………………………… |  |
| Seasonal vegetables ……………… |  |  | **Snacks** |  |
| Broccoli ……………………………….. |  |  | Twiglets……………………… |  |
| Green Beans ……………………. |  |  | __________________________ |  |
| Runner Beans ……………………….. |  |  |  |  |
| Brussel sprouts ……………………. |  |  |  |  |
| Potatoes ……………………………. |  |  |  |  |
| Spring greens ……………………… |  |  |  |  |
| Stringless Beans ……………………. |  |  |  |  |
| Cabbage (green) ……………………. |  |  |  |  |
| Cabbage (Savoy) ………………….... |  |  |  |  |
| Cabbage (White) ……………………. |  |  |  |  |
| Cauliflower …………………………… |  |  |  |  |
| Mangetout and sugarsnap peas …… |  |  |  |  |
| Mangetout ……………………………. |  |  |  |  |
| Sugarsnaps ………………………….. |  |  |  |  |
| Spinach ………………………………. |  |  |  |  |
| Mixed peppers ………………………. |  |  |  |  |
| Peppers (green) …………………….. |  |  |  |  |
| Peppers (red) ………………………… |  |  |  |  |
| Mixed frozen vegetables ………. |  |  |  |  |
